# A tripartite multifactorial analysis of abstract anaphoric choice between *this* and *this*+NP in Chinese L2 English argumentative writing: Extending the usage-based approach to discourse alternation

**DOI:** 10.1371/journal.pone.0347116

**Published:** 2026-04-17

**Authors:** Jianwei Xu, Ming Yue, Yujing Yang

**Affiliations:** Department of Linguistics, Zhejiang University, Hangzhou, Zhejiang, China; Father Muller Charitable Institutions, INDIA

## Abstract

The usage-based approach has documented L2 learners’ general convergence with native speakers in the probabilistic constraint patterns governing syntactic alternations. However, whether this convergence pattern extends to discourse-level phenomena remains an open question. This study addresses this gap by examining the abstract anaphoric alternation between *this* and *this*+NP (reduced vs. explicit form) in argumentative writing. We analyze a dataset of 1,304 instances from L2 students (Mandarin Chinese-L1) and 1,303 instances from a native English baseline. Each instance was annotated for four discourse-internal factors—*givenness*, *antecedent type*, *subjectness*, and *distance*—operationalizing the economy-clarity trade-off of accessibility theory. A tripartite multifactorial analysis—examining constraint significance, ranking, and interactions—was conducted within each of three sequential phases: first, establishing an L1 baseline model; second, independently characterizing the L2 system; and third, conducting a direct statistical comparison. Results indicate that: (1) L1 choices are systematically constrained by multiple probabilistic competing factors; (2) when analyzed independently, L2 learners show sensitivity to the same core constraints, constraint hierarchy, and fundamental interaction logic as native speakers; and (3) statistical comparison confirms broad convergence, with the only reliable divergence localized to a single context: L2 learners overuse the explicit *this*+NP in the highest-accessibility topic-continuity context. These findings demonstrate that L2 learners exhibit largely the same fundamental constraint system that guides native speakers’ choices, extending the scope of usage-based explanations from syntactic alternations to discourse-level phenomena in second language research.

## 1. Introduction

The usage-based approach views linguistic knowledge as probabilistically conditioned by frequent experience with language use [1,2], offering a distinctive theoretical and methodological perspective for studying linguistic alternation phenomena. Theoretically, this approach posits that the choice between alternative expressions is not arbitrary but is probabilistically shaped by multiple competing linguistic constraints that together determine a construction’s acceptability in context [3–5]. Methodologically, this probabilistic view has been advanced through multifactorial techniques that model how competing constraints collectively influence variant selection [6–8]. This set of constraints is understood to form part of speakers’ implicit knowledge, as predictions from corpus-based statistical models have been shown to align with speaker intuitions about variant acceptability [9,10].

Previous research applying this approach to L2 learners has focused primarily on syntactic alternations, including the dative alternation, genitive variation, *that*-complementation, and particle placement [6–8,11–13]. Across these phenomena, studies have generally found that L2 learners’ choices depend on a complex, interacting set of constraints similar to those that shape native speaker preferences. Where minor divergences emerge, they typically manifest as learners showing reduced sensitivity to certain processing-related constraints [6,13] and relying on fewer constraints overall than native speakers [11]. In other words, the core architecture of syntactic constraint systems appears to be largely shared between L1 and L2 speakers, with divergence appearing as limited, context-specific variations rather than fundamental differences in underlying knowledge.

While the usage-based approach has documented general L1-L2 constraint convergence in syntactic alternations, the question of whether similar dynamics operate at the discourse level remains open. Given that discourse-level choices are fundamentally shaped by universal functional pressures, such as information structure and topic management, one might reasonably expect strong convergence between L1 and L2 speakers in this domain as well. However, discourse phenomena differ from syntactic alternations in ways that may still pose challenges for L2 learners, even if the underlying functional logic is shared. Compared to syntactic alternations, discourse alternations are governed by constraints that span across sentence boundaries [14,15]. Their usage patterns are generally more implicit and less transparent in linguistic representation, characterized by form-function non-determinism and strong contextual dependency [16]. These properties require greater involvement of high-level abstraction and inferential mechanisms, thereby imposing more complex cognitive processing demands [17–19]. Given that L2 learners operate with limited processing resources and exhibit heightened sensitivity to processing constraints compared to native speakers [20,21], it is possible that even a fundamentally convergent system might exhibit subtle, localized divergences in the most demanding discourse contexts. Addressing this question offers one avenue for assessing the scope of usage-based explanations from syntactic studies to discourse in second language research.

Abstract anaphoric choice provides a suitable testing ground for this investigation. Abstract anaphora—reference to abstract entities, events, propositions, or states [22]—serves as a particular key device for signaling information structure and textual coherence [23]. Among others, demonstrative *this* is one of the most frequently used expressions in argumentative writing [24]. A tricky and persistent problem in the use of demonstrative *this* is the selection between the demonstrative pronoun *this* and the demonstrative determiner *this*+NP [24,25]. The former is typically used alone and represents a more reduced form, while the latter is usually attached to a noun or noun phrase, representing a more explicit form. It has been claimed that this choice is constrained by accessibility [26], representing a balance between economy and clarity [27–29], which can be represented by discourse-internal factors [30]. Thus, the appropriate choice of *this* and *this*+NP depends on the integration of nuanced discourse cues, making it possible to compare usage patterns of L1 and L2 speakers in this complex discourse domain.

Building on this foundation, the present study investigates L2 learners’ abstract anaphoric choice between *this* and *this*+NP. By analyzing usage patterns in authentic L2 argumentative writing and comparing them to those of native speakers, we examine whether L2 learners are sensitive to the same constraint patterns that influence native speakers’ choices, and to what extent.

## 2. Literature review

### 2.1. Choice of referring expressions in L1

Referential choice—the selection between reduced and explicit expressions (e.g., *this* vs. *this*+NP)—is a cornerstone of coherent discourse. A fundamental challenge in explaining language production lies in accounting for the frequent selection of reduced referring expressions where more explicit ones would ensure clarity [17]. Ariel’s accessibility theory resolves this paradox by positing that referential choice is guided by accessibility—a gradient property representing the cognitive status that a referent is presumed to have in the mental model of the reader [26,31]. The theory proposes a hierarchy where attenuated, low-informativity forms (e.g., *this*) signal highly accessible referents, while more rigid and informative expressions (e.g., *this*+NP) are required for less accessible entities [32]. Consequently, a speaker’s choice represents a strategic negotiation between the competing communicative imperatives of economy and clarity, aiming for optimal relevance without over-specification [27–29].

Empirical research has sought to identify the discourse-internal factors that realize accessibility, yet this effort has proceeded in a markedly unbalanced manner. A considerable body of work has successfully delineated factors under this theory influencing the choice for personal anaphora, such as recency, syntactic prominence, and givenness, among others [30]. In stark contrast, the exploration of factors under this framework to influence abstract anaphora—specifically, the alternation between *this* and *this*+NP—remains critically underexplored. To our knowledge, only a single corpus study by Botley and McEnery [33] has directly tested this alternation within an accessibility framework and found that referential distance—whether measured in words or in sentences—is the factor that influences the choice between *this* and *this*+NP.

While accessibility theory provides a powerful descriptive framework for the outcome of referential choice, it does not explain why particular language choices emerge or how multiple factors interact to shape these patterns. The usage-based approach effectively addresses this explanatory gap. This framework posits that language learning is a process of developing probabilistic weights for multiple competing linguistic motivators [1–5]. From this perspective, language choice is not random but reflects statistical learning and generalization that calculate constraint strength through exposure to language in use. Under this account, the factors highlighted by Ariel (e.g., recency, syntactic prominence, givenness) can be understood as regularities that language users extract from input through statistical learning. The usage-based approach thus complements accessibility theory by explaining how the discourse regularities that accessibility theory describes emerge from speakers’ statistical learning of the relationship between contextual cues and appropriate referring expressions.

This theoretical synthesis necessitates a methodological approach capable of modeling this probabilistic knowledge. A usage-based, multivariate analysis of corpus data is particularly well-suited for this task. By quantifying the relative weights and interactions of multiple factors, such analysis can reveal the statistical regularities that characterize speakers’ referential strategies. Thus, this study moves beyond testing the accessibility theory by providing an empirical model of how multiple constraints jointly influence language choice in naturalistic production.

In summary, previous research has yet to comprehensively identify the suite of discourse-internal factors influencing the choice of *this* and *this*+NP, let alone model their probabilistic competition and interaction. To address this empirical gap, the present study first identifies and operationalizes a set of theory-driven constraints hypothesized to influence this alternation. Using this taxonomy, it then constructs and analyzes the first multifactorial competition model for this phenomenon in L1 English. Establishing this L1 baseline is essential to provide both evidence for extending the usage-based approach to discourse and a benchmark for subsequent comparative analyses with L2 learner data.

### 2.2. Choice of referring expressions in L2

Research on L2 acquisition of anaphoric choice remains limited and has focused mainly on personal anaphora, leaving abstract anaphora largely unexamined. Within the study of personal anaphora, a consistent finding is the phenomenon of overexplicitness—learners’ tendency to overuse explicit referring forms in contexts where native speakers prefer reduced forms [15,34–37]. This divergence has been explained through three major theoretical accounts.

The interpretability hypothesis [38] attributes overexplicitness to a representational deficit, specifically the incomplete acquisition of L2-specific formal syntactic features. The interface hypothesis [39] locates the difficulty in the processing costs associated with integrating syntactic knowledge with discourse-pragmatic information. The pragmatic principles violation hypothesis (PPVH) [40] explains overexplicitness as a pragmatic safety strategy: learners tend to respect the principle of clarity (avoiding ambiguity) but frequently violate the principle of economy (providing redundant information) in order to avoid a communicative breakdown, especially in topic-continuity contexts [36].

While these accounts have advanced our understanding, their applicability to abstract anaphora is limited. The interpretability hypothesis and interface hypothesis are primarily concerned with constraints at syntax-related interfaces. However, the choice in abstract anaphora, such as *this* and *this*+NP, is less constrained by syntactic rules [41] and is instead governed by discourse-level constraints in terms of information structure and textual coherence [14,23]. Although the PPVH is the most relevant due to its focus on the economy-clarity trade-off and identifies a testable context (topic continuity) where L2 learners may struggle, its application has been largely restricted to personal anaphora and specific L1-L2 pairs (e.g., Spanish-English learners). More critically, it remains a descriptive account of the outcome rather than an explanation of how learners develop the probabilistic knowledge governing appropriate choice.

The usage-based approach effectively addresses this explanatory gap. A central premise of this framework is that both L1 and L2 learners develop linguistic knowledge through the same basic process: detecting patterns in the statistical regularities of the input—tracking frequencies and distributions, learning form-function associations, and abstracting generalizations from repeated encounters with language in use [2,3]. From this perspective, native speakers and L2 learners alike are understood to internalize probabilistic constraints through the competition model, gradually learning to weight multiple discourse cues. Given that the actual shape of the input language is roughly the same for both groups [2,3], convergence is the default expectation: with sufficient exposure, L2 learners should develop constraint sensitivities broadly similar to those of native speakers.

While convergence is the default expectation, the conditions of learning differ in ways that may lead to localized divergence. L2 learners often face quantitative and qualitative differences in input compared to native speakers [2,3]. Quantitatively, they typically receive less extensive exposure to the target language. Qualitatively, their input is filtered through an already-established L1 system and lacks the intensive social support that characterizes first language acquisition—the one-on-one interaction, scaffolded feedback, and immersive engagement. These differences can result in weaker associations between specific linguistic cues and language choices, making certain constraints more difficult to process [20,21,42,43]. This can lead L2 learners to adopt conservative strategies, which may result in overgeneralizations or simplifications [6,13]. A key testable prediction is that this learning scenario manifests as the well-documented overexplicitness in topic-continuity contexts—precisely the phenomenon described by the PPVH [40], but now provided with a theoretical explanation.

However, to date, no study has examined L2 learners’ sensitivity to probabilistic constraints in abstract anaphoric choice. Filling this gap allows us to move beyond descriptive and test whether L2 learners exhibit the general convergence constraint patterns as native speakers, and whether any divergence is limited to specific discourse contexts.

### 2.3. The present study

This study investigates the choice between abstract anaphoric *this* and *this*+NP in argumentative writing. We analyze a dataset balanced by the number of instances per group: 1,304 instances from L2 students and 1,303 instances from a native English baseline. We focus on L2 learners with Mandarin Chinese as an L1. This population is well-suited because Mandarin possesses a parallel proximal demonstrative system (*zhe* versus *zhe* + NP) [44,45], presenting learners with a structurally similar choice and reducing the likelihood of overt transfer effects at the morphosyntactic level, though more subtle discourse-level influences cannot be entirely ruled out. This paper is guided by two research questions:

(1) How do multiple competing discourse-internal factors interact to predict the anaphoric choice between *this* and *this*+NP in L1 English writing?
(2) To what extent do L2 learners demonstrate target-like probabilistic patterns for this alternation?

By addressing these questions, this study makes three primary contributions. First, it establishes a systematic taxonomy of discourse-internal factors that predict the choice between *this* and *this*+NP, thereby addressing a fundamental gap in empirical literature. Second, it develops and validates the first multifactorial competition model for abstract anaphora, thereby extending the explanatory scope of the usage-based approach to complex discourse phenomena. Third, by comparing this model independently and integrally with L2 data, it tests the extent to which L2 learners exhibit probabilistic constraint patterns similar to those of native speakers, offering a systematic comparison of convergence and divergence in discourse-level production.

Importantly, while our data can reveal whether learners show similar patterns of constraint sensitivity, they cannot directly speak to the processes by which such knowledge is acquired; rather, they provide evidence about the outcome of learning as reflected in end-state production.

## 3. Methodology

### 3.1 The corpora

Guided by the usage-based approach, this study investigates the probabilistic model of abstract anaphoric choice and its consistency across L1 and L2 writers. To enable a rigorous comparison, a specialized corpus was compiled for maximal comparability, consisting of two principal subcorpora: argumentative essays by Chinese learners of English (L2 corpus) and a baseline of comparable texts by native English speakers (L1 corpus).

The methodological selection of the argumentative genre is principled, adhering to core tenets of corpus design. This choice is paramount for its contextual representativeness; demonstrative *this* ranks among the most frequently used expressions in argumentative writing [24], a genre whose core communicative goal is to construct logically coherent discourse by systematically linking abstract propositions [46]. Studying the choice between *this* and *this*+NP within this context ensures it is observed in its highly naturalistic and ecologically valid environment. Furthermore, this selection ensures corpus homogeneity, a critical prerequisite for a controlled L1-L2 comparison, by systematically controlling for the documented influence of genre variation on anaphoric choice [46–48].

The L2 corpus was sourced from a principled compilation of two authoritative, open-access corpora of Chinese EFL writers: the Written English Corpus of Chinese Learners (WECCL) [49] and the Ten-thousand English Compositions of Chinese Learners (TECCL) [50]. Together, these corpora provide a substantial collection of argumentative essays across a diverse range of Chinese universities, ensuring a representative sample of L2 written production.

The L1 baseline was established using the Louvain Corpus of Native English Essays (LOCNESS) [51], a benchmark resource in learner corpus research. To ensure maximal comparability with the L2 data, the analysis draws exclusively from the argumentative essays within the British (BRSUR3) and American (USARG) subsections of LOCNESS, which feature writing from university English native students of comparable writing genre. These two subcorpora were combined to form a unified native-speaker baseline. This decision is theoretically grounded, as the abstract anaphoric alternation is governed by discourse-pragmatic principles (e.g., information structure) rather than by features that vary systematically between British and American English. Consequently, this combined baseline provides a more robust and representative L1 model, enhancing the generalizability of the findings.

All source corpora are publicly available for academic scrutiny:

(1) (WECCL: https://discx.yuntu.io/disc/7679804073959;
(2) TECCL: https://corpus.bfsu.edu.cn/info/1070/1449.htm;
(3) LOCNESS: https://www.learnercorpusassociation.org/resources/tools/locness-corpus/).

The use of these pre-existing, fully anonymized datasets exempted this study from further ethics review and the requirement for informed consent.

A uniform data processing protocol was implemented across all texts. Every instance of the demonstrative *this* and *this*+NP was extracted. Each token was then manually disambiguated to isolate genuine anaphoric references from non-anaphoric uses. Texts that contained no instances of anaphoric *this* and *this*+NP were excluded from the final dataset, ensuring all analyzed texts contributed relevant data. This procedure yielded a final dataset of 2,607 anaphoric instances (L2: 1,304; L1: 1,303).

In response to reviewer requests for transparency regarding participant‑level information and subject‑level variability, we report the following detailed distributions: the L2 instances originated from 568 unique writers (mean = 2.30, median = 2, SD = 1.55, IQR = 1–3, range = 1–9), while the L1 instances came from 194 unique writers (mean = 6.72, median = 5, SD = 6.16, IQR = 3–9, range = 1–44). This detailed accounting—particularly the low medians (L2: 2; L1: 5), narrow interquartile ranges (L2: 1–3; L1: 3–9), and the high number of unique writers (L2: 568; L1: 194)—confirms that the data, while imbalanced, is not unduly dominated by a small number of prolific writers and supports the generalizability of the findings.

A notable characteristic of the source corpora is that the L1 essays are, on average, longer than the L2 essays (mean word count: L1 = 833, L2 = 440). This difference in text length contributes to the higher mean number of instances per writer in the L1 corpus. In this corpus structure, each writer contributed a single text, creating a hierarchical data structure where observations (individual instances of *this*/*this*+NP) are nested within writer/text units. This nesting creates statistical non‑independence, as instances from the same writer or text may be more similar to each other than to instances from other sources [52].

Our analytical design is explicitly constructed to address this structure and ensure a valid comparison. The fundamental unit of analysis is the individual instance of potential anaphoric choice. We model the conditional probability of selecting *this* versus *this*+NP at each comparable decision point—a focus that is logically independent of the raw frequency of such points per text. Furthermore, our tripartite analytical strategy (Section 3.3) employs methods robust to hierarchical and imbalanced data. Consequently, while the source texts differ in average length, our research directly targets the probabilistic constraints governing referential choice where the alternation is possible, ensuring a methodologically sound L1‑L2 comparison.

### 3.2 Operationalization and annotation

The operationalization of the potential factors was theoretically grounded in accessibility theory [26,31], which posits that referential choice is constrained by the referent’s cognitive accessibility. The dependent variable was the binary choice between the anaphoric forms *this* and *this*+NP. We investigated four discourse-internal independent factors: *distance*, *subjectness*, *antecedent-type*, and *givenness*, alongside the external variable *nativeness* (L1 vs. L2 writer).

The specific internal factors were selected because of their theoretical relevance to the information-structural management of abstract referents in prior theoretical and empirical work [14,23,25,31,33]. Factors influential for personal anaphora (e.g., animacy) were not considered, as they are less pertinent to non-human, abstract referents. The external factor *nativeness* was included to address the study’s comparative focus. To further control for the influence of other external factors such as register and genre variation on anaphoric choice [46–48], the present study restricts its focus to a single, well-defined register—argumentative writing—as detailed in Section 3.1.

The annotation scheme for each factor is detailed below.

**Distance:** This factor measures how far an antecedent is from its anaphor and serves as a potential influential factor of accessibility [26]. In corpus-based studies, sentence-based distance is a more reliable measure than word count because structural proximity between an antecedent and an anaphor directly affects referent activation [53]. Moreover, since abstract anaphors frequently refer to lengthy non-nominal antecedents, calculating distance in words may yield less meaningful results [54]. Therefore, measuring referential distance in sentences provides a more accurate representation of accessibility dynamics.

We employed a binary classification: *previous* and *far*. This classification follows [54] but was adapted to better capture the prevalence of long-distance anaphora in abstract contexts [14,18]. Specifically, referents located within the same sentence or the immediately preceding sentence were annotated as *previous* (see Example 1); referents spanning two or more sentences away were annotated as *far* (see Example 2). A unified rule was applied: *distance* was measured from the anaphor to the first sentence of the antecedent span. This rule ensures consistent treatment for all antecedent types—whether a single phrase or a multi-sentence unit, capturing the recency of referent initiation as a key determinant of accessibility.

(1) When offered these facts some try to suggest that we eliminate the appeal system [distance: previous]. ***This approach*** is impractical because the appeal system is a major part in the backbone of our judicial system. < Native-USA-SCU-0011.4.txt>
(2) In this movement poorhouses were established [distance: far]. As with the cheap labor, poorhouses were only available to the people that were the most “deserving” of the poor. ***This attempt*** did not help prevent homelessness. < Native-USA-PRB-0037.2.txt>

**Subjectness:** The syntactic function of anaphors, whether they appear in subject or non-subject positions, is closely tied to the notion of saliency. It serves as a reliable and easily measurable syntactic proxy for discourse relevance and has been widely used in experimental and corpus-based studies on referential expression variation, particularly in distinguishing between nominal and pronominal expressions [53,54].

We employed a binary classification: *subject* (as in Example 3) and *non-subject* (as in Example 4) [24].

(3) As I have said, sovereignty will be lost but, with an innovative and unblinkered view. ***This*** [subjectness: subject] could be a positive rather than negative development for Britain. < Native-BRS0005.3.txt>
(4) For example, a star gave birth to a girl who was born with a harelip. The mass media bombed ***this “big event”*** [subjectness: non-subject] immediately despite the parents’ feeling and future development of the girl. < Nonnative-WARG1832.txt>

**Antecedent-type:** The syntactic form of the antecedent was annotated based on its established relationship with semantic richness and accessibility [55]: Non-nominal antecedents, typically realized by clauses or larger units, are longer and semantically richer, often favoring reduced anaphoric forms. In contrast, nominal antecedents, realized as nouns or noun phrases, are typically shorter and more likely to be referred to with explicit forms. Demonstratives, which can refer to both types, are thus crucial for investigating this claim.

We performed a binary classification into *non-nominal* and *nominal* antecedents. This annotation follows [18], where the former refers to clausal or larger syntactic units, as in Example (5), while the latter refers to nouns or noun phrases, as in Example (6). In instances where the referent could be interpreted as either a nominal phrase or a larger clausal unit, annotators applied a rule of semantic substitutability: the antecedent was coded as *nominal* only if the anaphor could be semantically replaced by the noun phrase alone without altering the propositional meaning of the utterance. This ensured consistent resolution of ambiguous cases based on the semantic scope of the anaphor.

(5) One must consider what it would be like for one woman to share a barrack with nearly one hundred men, or vice versa [antecedent-type: non-nominal]. ***This*** would be a very uncomfortable situation for whomever. < Native-USA0013.1.txt>
(6) The political and economic exhaustion of the European states called for a fresh start [antecedent-type: nominal] and a far more radical approach to the re-ordering of Europe. ***This fresh start*** came about because of three main factors. < Native-BRS0004.3.txt>

**Givenness:** This factor captures whether the anaphoric expression provides additional semantic information not present in its antecedent, a key feature influencing topic management and discourse coherence [23]. This operational definition is supported by functional linguistic research indicating that both *this* (by the *this*+is + NP structure) and *this*+NP possess the potential to introduce new information [24,41,56]. Functionally, when the anaphor in subject position supplies only given information, it signals topic continuity, maintaining the ontological status of the referent. Conversely, when it introduces new information, the anaphor in subject position marks topic shift, redefining the referent through evaluation or classification and establishing a new discourse focus.

This functional distinction aligns with accessibility theory [26,31] and empirical findings on coherence relations [57]: higher topic continuity correlates with higher accessibility, favoring the reduced anaphoric form, whereas topic shift reduces accessibility and motivates the more explicit anaphoric form. Thus, the presence or absence of new information in the anaphor not only reflects local coherence but also probabilistically predicts the choice between anaphoric forms.

The factor was annotated on a binary scale: *given* and *new*. This dichotomous operationalization relates to information structure as noted in the prior paragraph, distinguishing anaphors that provide no new nominal information from those that introduce a novel nominal predicate. Operationally, the categories were defined as follows:

The *given* category was assigned when the anaphor provided no new information, encompassing bare *this* (see Example 7) and *this*+NP where the attending noun is lexically or morphologically recoverable from the antecedent (see Example 8).

(7) The children grow up watching violence and sex and they become desensitized to it. ***This*** [givenness: given] creates problems with values which in turn creates problems in society. < Native-USA-MICH-0016.1.txt>
(8) They are nothing but a tool. ***This tool*** [givenness: given] is used when you are not convenient to use a traditional one. < Nonnative-WARG2963.txt>

The *new* category was assigned when the anaphor introduced a novel nominal concept. A crucial clarification is that this category does not indicate the expression is non-anaphoric; all analyzed instances are anaphoric by design. Instead, *new* is assigned specifically when a novel nominal concept is introduced to characterize the existing referent, as noted in the prior paragraph. For bare *this*, this was only possible in the specificational structure “*this*+is+NP”, where the post-copular NP provides new predicative information about the anaphoric referent (see Example 9). For *this*+NP, the new category was assigned when the head noun had not appeared in the antecedent in any form (see Example 10).

(9) How about making the paycheck out to the couple, or to the entire family, instead of the individual person? Perhaps ***this is a solution*** [givenness: new] worth its salt since it communicates physically, materially, that each member of a partnership is an equally worthy contributor. < Native-USA-IND-0004.1.txt>
(10) For example, a star gave birth to a girl who was born with a harelip. The mass media bombed ***this “big event”*** [givenness: new] immediately despite the parents’ feeling and future development of the girl. < Nonnative-WARG1832.txt>

The author first developed a preliminary annotation scheme and consulted linguistic experts to discuss uncertain cases until a consensus was reached. Subsequently, the author annotated four discourse-internal factors within the entire corpus, while an independent Ph.D. student in linguistics annotated a randomly selected 20% of the instances. Inter-annotator reliability was assessed using Cohen’s Kappa [58], with values below 0.40 indicating poor agreement, values between 0.40 and 0.75 indicating fair to good agreement, and values above 0.75 indicating excellent agreement. All four discourse-internal factors in this study were assessed, yielding values of 0.76 for *distance*, 0.79 for *antecedent-type*, 0.86 for *givenness*, and 0.93 for *subjectness*—each exceeding the 0.75 threshold and thus indicating excellent inter-annotator agreement.

### 3.3 Data analysis

Aligned with the core tenets of the usage-based approach [1], this study employs authentic corpus data and multifactorial analysis to model the probabilistic constraints governing linguistic choice. To this end, we implemented a tripartite analysis examining three distinct yet interrelated dimensions of the constraint system: statistical significance, predictor ranking, and complex interactions. These dimensions were addressed using two complementary methods: mixed-effects logistic regression and conditional inference trees (CIT)—which were selected for their complementary strengths and their suitability for the hierarchical structure and imbalanced distributions of naturalistic corpus data (see Section 3.1).

Mixed-effects logistic regression [52] determines which predictors exert a statistically reliable influence on a binary linguistic choice (significance) and whether that influence favors one variant over the other (direction). It does so by estimating the effect of each predictor while accounting for the non‑independence of observations via random intercepts for corpus items (e.g., texts, speakers), directly addressing the hierarchical structure of naturalistic data.

CIT [59] addresses the remaining two dimensions. First, they provide a transparent ranking of predictor importance through their hierarchical splitting order: variables that appear earlier in the tree exert stronger constraints on the outcome. Second, they visualize how multiple factors interact to predict a binary outcome by recursively partitioning the dataset using a sequence of statistical hypothesis tests. At each step, the algorithm identifies the predictor and split point that yields the most significant separation between the two categories, continuing until no further significant splits remain. Because splits are based on conditional inference tests, CIT avoids variable selection bias common in other tree‑based methods and provides statistically valid partitions.

Together, these methods form a complete analytical toolkit. Regression establishes which constraints matter and in what direction; CIT reveals both how much they matter (through ranking) and the specific discourse contexts in which their influence takes effect (through interaction visualization). This multi‑perspective design ensures that findings about constraint competition are robustly supported across complementary statistical lenses.

All analyses were conducted in *R* 4.4.2 [60] using the *lme4* package for mixed-effects modeling [61], and the *ctree* function for generating conditional inference trees [59].

The analysis proceeded in three sequential phases, each building directly on the previous one.

Phase 1: L1 Baseline Model. This phase defined the target constraint probabilistic model in native English writing using the L1 dataset (n = 1,303). Our analysis began with a mixed-effects logistic regression model, fitted with the binary anaphoric choice as the dependent variable and the four discourse-internal factors as fixed effects. The model incorporated random intercepts for corpus items to mitigate biases from repeated measures and hierarchical data structure. Prior to modeling, collinearity diagnostics were conducted using Pearson correlation coefficients, and the model’s classification quality was confirmed with a *C*-index value above 0.8, indicating a good fit [52]. CIT analysis was then conducted to both rank predictor importance and visualize interactions among constraints. Together, these analyses establish the L1 constraint system as a stable, interpretable benchmark.

Phase 2: Independent L2 Model. This phase applied the identical tripartite protocol—significance, ranking and interaction—exclusively to the L2 dataset (n = 1,304). Its purpose was to determine whether a systematic probabilistic grammar governs L2 choices on its own terms, prior to any comparison with the L1 baseline. The L2-specific regression identified significant constraints and their effect sizes; the L2-specific CIT established the internal constraint hierarchy and uncovered interaction patterns unique to learner production. This phase ensures that L2 knowledge is characterized as a coherent system in its own right.

Phase 3: Comparative L1-L2 Analysis. Building directly on the independently specified L1 and L2 systems, this final phase integrated both datasets (n = 2,607) to conduct direct statistical tests of convergence and divergence. The regression model included *nativeness* as a fixed effect and tested its interactions with the four discourse-internal factors. The CIT evaluated whether *nativeness* emerged as a significant splitting variable within the combined constraint hierarchy and whether it interacted with other factors to create distinct discourse contexts. This phase provides the inferential evidence needed to determine whether observed cross-group similarities and differences are statistically reliable.

This phased workflow—from independent system description to direct statistical comparison—forms a complete analytical chain that provides both descriptive rigor and inferential power. This multi‑perspective, multi‑phase framework ensures that findings regarding constraint competition are convergently supported across complementary analytical lenses and are not artifacts of a single modeling assumption.

## 4. Results

This section presents the statistical findings in three parts. Section 4.1 establishes the baseline probabilistic model of constraints governing abstract anaphoric choice in L1 English writing. Section 4.2 presents the independent L2 model, characterizing the learner constraint system on its own terms. Section 4.3 then reports the comparative L1-L2 analysis, statistically evaluating the extent to which L2 patterns converge with or diverge from the native baseline.

### 4.1 L1 baseline model

**Fig 1:**
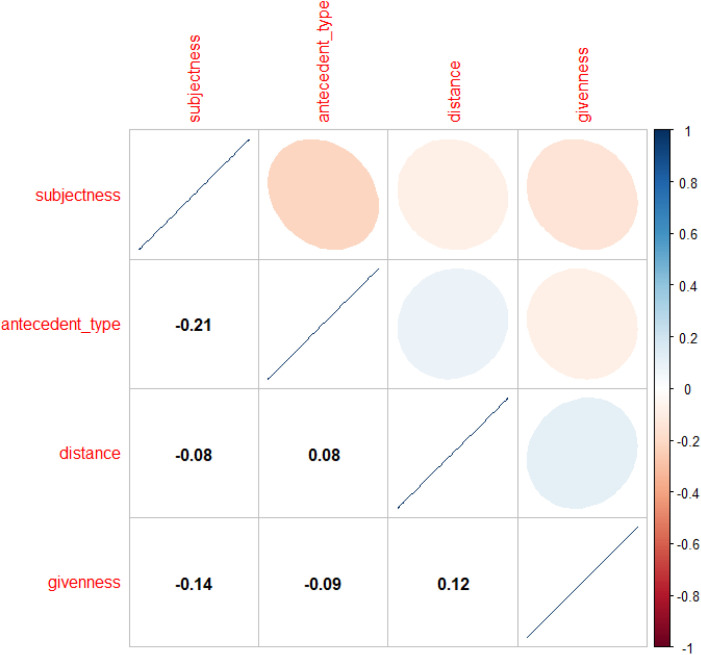
Correlation matrix of fixed effects in the regression model for abstract anaphoric choice in L1 dataset.

The analysis of the native English corpus (n = 1,303) began with a mixed-effects logistic regression model predicting the binary choice between *this* (coded as 0) and *this*+NP (coded as 1). Before model estimation, collinearity diagnostics using Pearson correlation coefficients confirmed no significant multicollinearity among the factors (*r* < 0.8), ensuring model stability (see **Fig 1**).

To examine how anaphoric choice was affected by different discourse-internal factors, we analyzed anaphoric choice (*this* vs. *this*+NP) as a function of *distance*, *subjectness*, *antecedent-type*, and *givenness*. The model shows a significant correlation between anaphoric choice and the factors (*p* < 0.001, pseudo *R*^*2*^ = 0.57, *C* = 0.89). The *C*-value is 0.89, which is higher than 0.8, indicating a good fit to the data. The statistics also show that 82.43% of anaphoric choices are classified correctly, which is significantly higher than a chance accuracy of 56.25%. Chance accuracy is the majority class baseline, calculated as the proportion of the most frequent anaphoric form in the analyzed dataset. In the L1 corpus, *this*+NP is the majority form (733 of 1,303 instances), yielding a baseline of 56.25%. Full frequency distributions are available in the supporting information file (S1 File.xlsx), ensuring complete transparency of all descriptive statistics underlying these calculations.

As detailed in Table 1, the analysis revealed significant main effects for all four discourse-internal factors. The direction of the coefficients indicates that native speakers are significantly more likely to choose the explicit form *this*+NP when the anaphor is in a non-subject position, refers to a nominal antecedent, appears at a long distance, or provides new information.

**Table 1:** Summary of the mixed-effects logistic regression models for abstract anaphoric choice in L1 dataset.

Fixed effects	b	Lower CI	Upper CI	Odds ratio	p	
(Intercept)	−1.09	−1.40	−0.78	0.34	<0.001	***
subjectness (subject)	−1.36	−1.67	−1.05	0.26	<0.001	***
antecedent-type (nominal)	2.40	2.02	2.78	11.03	<0.001	***
distance (far)	0.42	0.07	0.77	1.52	0.018	*
givenness (new)	2.98	2.62	3.34	19.74	<0.001	***

**Fig 2:**
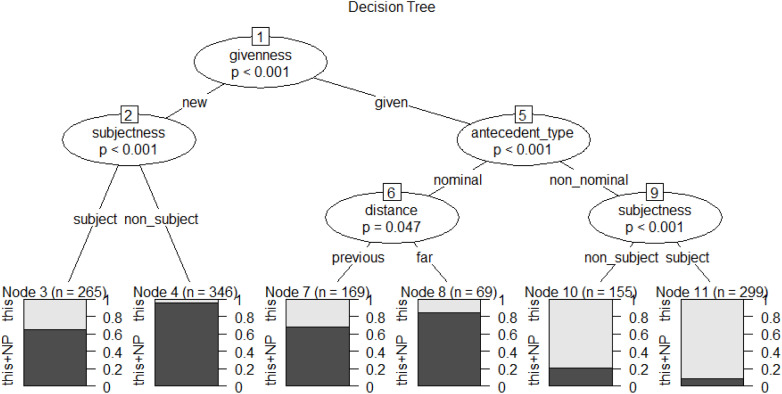
Conditional inference tree for abstract anaphoric choice in L1 dataset.

To establish a transparent ranking of their relative strength and visualize the complex interactions among the significant factors, we constructed conditional inference trees. The resulting model for the anaphoric choice between *this* and *this*+NP (see Fig 2) achieved a classification accuracy of 82.34%, substantially exceeding the baseline accuracy of 56.25% and demonstrating strong predictive power.

In Fig 2, the elliptical nodes represent independent variables with statistically significant effects on the dependent variable, each labeled with node numbers at the top. Predictors appearing at higher hierarchical levels in the tree structure indicate a stronger influence on the dependent variable. Branches represent splitting conditions determined by statistical significance between predictors and outcome, executing recursive binary partitioning until no significant splits remain [59]. Terminal leaf nodes (bottom positions) represent final data subsets meeting combined splitting conditions, annotated with node numbers and total cases. Light/dark gray proportions indicate relative frequencies of anaphoric *this* vs. anaphoric *this*+NP.

As shown in Fig 2, the CIT analysis corroborates the significant influence of all four discourse factors, with their hierarchical position within the tree structure providing a visual representation of their relative predictive strength. The resulting constraint hierarchy, from the strongest to weakest, is: *givenness*, *antecedent-type*, *subjectness*, and *distance*. Based on the splitting patterns of these four factors, we identified six split interaction conditions. Table 2 organizes these contexts in descending order of the proportion of anaphoric *this* usage.

**Table 2:** The split conditions of conditional inference tree for abstract anaphoric choice in L1 dataset.

Context	Split Conditions	Node	*This*	*This*+NP	Total
1	given + non-nominal + subject	11	92%	8%	100%
2	given + non-nominal + non-subject	10	79%	21%	100%
3	new + subject	3	35%	65%	100%
4	given + nominal + previous	7	32%	68%	100%
5	given + nominal + far	8	16%	84%	100%
6	new + non-subject	4	4%	96%	100%

**Context 1 (Node 11):** Characterized by the co-occurrence of given information, a non-nominal antecedent, and subject position, this combination represents the prototypical topic-continuity context for abstract propositions, events, or states, yielding the highest accessibility level, thus resulting in the highest probability of reduced *this* (92%).

**Context 2 (Node 10):** When the anaphor occupies a non-subject position, the accessibility is attenuated, resulting in a corresponding decrease in the selection of reduced *this* (79%).

**Context 3 (Node 3):** The introduction of new information precipitates a shift towards the explicit *this*+NP, even when other features, such as subject position, might otherwise support a reduced form (35%).

**Context 4 (Node 7) and Context 5 (Node 8):** A nominal antecedent type exerts a strong constraining effect, favoring explicit *this*+NP. A gradient effect of referential distance is evident, where previous distance (32%) corresponds to a higher likelihood of the reduced form than far distance (16%).

**Context 6 (Node 4):** The confluence of new information and non-subject position constitutes the lowest accessibility context, resulting in the lowest probability of selecting the reduced *this* (4%).

In summary, the analysis of the L1 dataset indicates that *givenness*, *antecedent-type*, *subjectness*, and *distance* all significantly constrain the choice between anaphoric *this* and *this*+NP, with constraint ranking descending in that order. Furthermore, the interaction of these factors yields six distinct contexts. Among these, the confluence of three high-accessibility levels—given information, non-nominal antecedent, and subject position—constitutes the highest accessibility context (topic-continuity), which is associated with the highest probability of selecting the reduced form *this*.

### 4.2 Independent L2 model

Having established the L1 baseline, we now turn to the L2 system, analyzing it independently before any direct comparison. This approach allows us to characterize learner knowledge on its own terms, revealing whether a systematic probabilistic grammar governs L2 choices and how its internal structure descriptively aligns with or diverges from the native benchmark.

**Fig 3:**
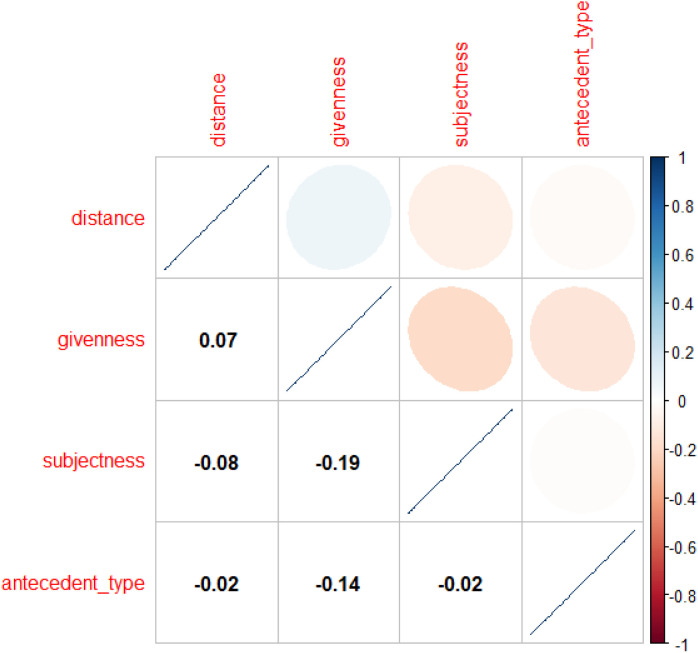
Correlation matrix of fixed effects in the regression model for abstract anaphoric choice in L2 dataset.

The analysis of the L2 English corpus (n = 1,304) began with a mixed-effects logistic regression model predicting the binary choice between *this* (coded as 0) and *this*+NP (coded as 1). Before model estimation, collinearity diagnostics using Pearson correlation coefficients confirmed no significant multicollinearity among the factors (*r* < 0.8), ensuring model stability (see **Fig 3**).

To examine how anaphoric choice was affected by different discourse-internal factors, we analyzed anaphoric choice (*this* vs. *this*+NP) as a function of *distance*, *subjectness*, *antecedent-type*, and *givenness*. The model shows a significant correlation between anaphoric choice and the factors (*p* < 0.001, pseudo *R*^*2*^ = 0.61, *C* = 0.93). The *C*-value of 0.93 exceeds 0.8, indicating a good fit to the data. The model correctly classified 85.43% of anaphoric choices, substantially exceeding the baseline chance accuracy of 69.40%—the proportion of the majority class (*this*+NP, which accounted for 905 of 1,304 instances) in the L2 dataset. Full frequency distributions are available in the supporting information file (S1 File.xlsx).

As detailed in Table 3, the analysis revealed significant main effects for three of four discourse-internal factors. The direction of the coefficients indicates that L2 speakers are significantly more likely to choose the explicit form *this*+NP when the anaphor is in a non-subject position, refers to a nominal antecedent, or provides new information. *Distance*, however, did not exert a significant influence on L2 choice.

**Table 3:** Summary of the mixed-effects logistic regression models for abstract anaphoric choice in L2 dataset.

Fixed effects	b	Lower CI	Upper CI	Odds ratio	p	
(Intercept)	−0.53	−0.86	−0.20	0.59	0.002	**
subjectness (subject)	−1.90	−2.29	−1.51	0.15	<0.001	***
antecedent-type (nominal)	2.22	1.65	2.80	9.25	<0.001	***
distance (far)	0.20	−0.17	0.57	1.22	0.284	
givenness (new)	3.39	2.89	3.89	29.74	<0.001	***

Comparing Table 3 with the L1 model (Table 1) reveals descriptive convergence on three core factors (*subjectness*, *antecedent-type*, *givenness*) in both significance and direction. The only descriptive difference is *distance*, significant for L1 but not L2. The integrated analysis (Section 4.3) will test these patterns statistically.

**Fig 4:**
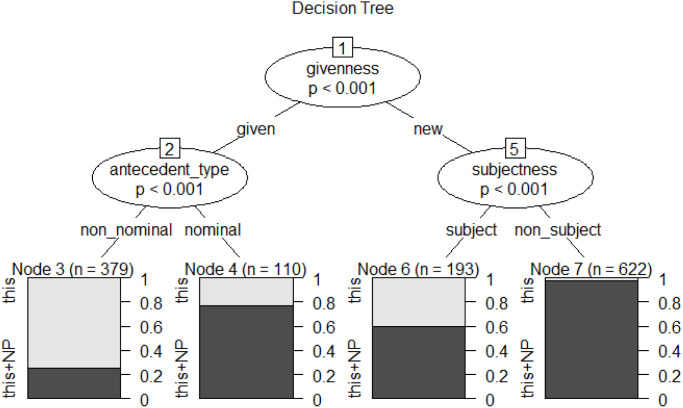
Conditional inference tree for abstract anaphoric choice in L2 dataset.

To establish a transparent ranking of the relative strength and visualize the complex interactions among the significant factors, we constructed conditional inference trees. The resulting model for the anaphoric choice between *this* and *this*+NP (see Fig 4) achieved a classification accuracy of 83.59%, substantially exceeding the baseline accuracy of 69.40% and demonstrating strong predictive power.

As shown in Fig 4, the resulting constraint hierarchy, from strongest to weakest, is: *givenness*, *antecedent-type*, *subjectness*—a ranking that is descriptively identical with that of the L1 baseline.

Based on the splitting patterns of these three factors, we identified four interaction contexts. Table 4 organizes these contexts in descending order of the proportion of anaphoric this usage.

**Table 4:** The split conditions of conditional inference tree for abstract anaphoric choice in L2 dataset.

Context	Split Conditions	Node	*This*	*This*+NP	Total
1	given + non-nominal	3	74%	26%	100%
2	new + subject	6	40%	60%	100%
3	given + nominal	4	23%	77%	100%
4	new + non-subject	7	2%	98%	100%

Comparison with L1 (Table 2) reveals shared fundamental logic: *givenness* partitions the data first, followed by *antecedent-type* within the *given* branch and *subjectness* within the *new* branch. However, L2 shows less granularity at deeper levels. Under the *antecedent-type* split, L2 has no further division: the *nominal* branch lacks the *distance* split found in L1, and the *non-nominal* branch lacks the *subjectness* split found in L1. These descriptive differences require statistical confirmation.

The independent L2 analysis reveals a system strongly convergent with L1: learners show sensitivity to the same core constraints, the same directional effects, the same constraint hierarchy, and the same fundamental interaction logic. Descriptive divergences are limited to the non-significance of *distance* and reduced granularity at the deepest interaction levels. Section 4.3 tests whether these descriptive patterns—both the convergences and the divergences—are statistically reliable.

### 4.3 Comparative L1-L2 analysis

Having characterized the L1 and L2 systems independently, we now integrate both datasets to test statistically whether the descriptive patterns of convergence and divergence are statistically reliable. The combined dataset (n = 2,607) was analyzed with *nativeness* included as an additional factor, allowing direct tests of group differences and their interactions with the discourse-internal constraints.

The mixed-effects logistic regression model shows a significant correlation between anaphoric choice and the factors (*p* < 0.001, pseudo *R*^*2*^ = 0.58, *C* = 0.90). The *C*-value is higher than 0.8, indicating a good fit to the data. The statistics also show that 83.28% of anaphoric choices are classified correctly, which is significantly higher than a chance accuracy of 63.83%—the proportion of the majority class (*this*+NP, which accounted for 1,638 of 2,607 instances) in the combined dataset. Full frequency distributions are available in the supporting information file (S1 File.xlsx).

As shown in Table 5, all four discourse-internal factors remain significant with the same directional effects as in independent L1 and L2 models. A significant main effect of *nativeness* indicates that L2 learners have a higher overall probability of choosing *this*+NP.

**Table 5:** Summary of the mixed-effects logistic regression models for abstract anaphoric choice in L1-L2 datasets.

	b	Lower CI	Upper CI	Odds ratio	p	
(Intercept)	−0.61	−0.85	−0.38	0.54	<0.001	***
nativeness (native)	−0.33	−0.58	−0.09	0.72	0.008	***
subjectness (subject)	−1.58	−1.81	−1.35	0.21	<0.001	***
antecedent-type (nominal)	2.30	1.99	2.61	9.93	<0.001	***
distance (far)	0.31	0.06	0.55	1.36	0.014	*
givenness (new)	3.06	2.79	3.34	21.43	<0.001	***

**Fig 5:**
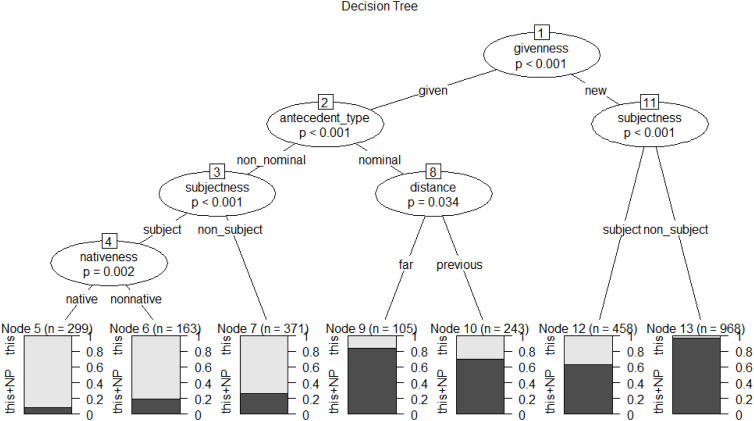
Conditional inference tree for abstract anaphoric choice in L1-L2 datasets.

To examine constraint ranking and interactions including *nativeness*, we constructed conditional inference trees on the combined dataset. Fig 5 presents the resulting model, achieving a classification accuracy of 82.97%, which exceeds the baseline accuracy of 62.83%, indicating strong classificatory and predictive power.

As shown in Fig 5, the CIT analysis yields three key findings. First, the constraint hierarchy, from the strongest to weakest, is: *givenness*, *antecedent-type*, *subjectness*, *nativeness,* and *distance*—with *nativeness* ranking below the three primary discourse factors, indicating that group membership modulates choice only after the core discourse constraints have applied.

Second, and critically, the analysis reproduces the same six interaction contexts previously identified in the L1 baseline. This overall structure provides strong evidence that the fundamental architecture of the constraint system is shared across L1 and L2 writers. Table 6 presents these contexts in descending order of the proportion of *this* usage.

**Table 6:** The split conditions of conditional inference tree for abstract anaphoric choice in L1-L2 datasets.

Context	Split Conditions	Node	*This*	*This*+NP	Total
1	given + non-nominal + subject	5, 6	86%	14%	100%
2	given + non-nominal + non-subject	7	74%	26%	100%
3	new + subject	12	37%	63%	100%
4	given + nominal + previous	10	30%	70%	100%
5	given + nominal + far	9	16%	84%	100%
6	new + non-subject	13	3%	97%	100%

Third, within this shared architecture, *nativeness* creates a significant split (*p* < 0.002) in exactly one context: the highest-accessibility topic-continuity context (given + non-nominal + subject), as indicated by elliptical Node 4 “nativeness” in Fig 5. Here, L2 writers use explicit *this*+NP at a significantly higher rate than native speakers, even though both groups prefer the reduced form overall. Importantly, this divergence is not a breakdown of the shared architecture, but rather a systematic quantitative difference within a context where the qualitative constraint logic is already in place. The fact that *nativeness* emerges as a split only at the deepest level of the tree—and only within the highest-accessibility context—underscores that the divergence is localized rather than systemic.

This divergence is exemplified in the following instances from this specific context. Given the high accessibility of this context, native speakers consistently select the reduced *this* to maintain topic continuity for abstract propositions efficiently (Examples 11–12). L2 learners in the same context also show a preference for reduced *this*, but their usage is characterized by a significantly higher rate of the explicit *this*+NP (Examples 13–14), suggesting a reduced sensitivity to the pragmatic redundancy of overt reference when accessibility is maximal.

(11) These students need knowledge and skills to protect themselves and their partners. ***This*** means that explicit instruction in what types of sex are most risky and how to reduce risk by using condoms is necessary. < Native-USA0021.1.txt>
(12) If there were fewer civil cases to be dealt with, it would free up courts to deal with more criminal cases, reducing the backlog of criminal cases, which would bring the criminal cases to trial quicker. ***This*** would result in fewer criminals out on bond, as well as fewer inmates in county jails awaiting trial. Bringing the criminal cases to trial quicker would reduce overcrowding in local jails. < Native-USA-SCU-0016.4.txt>
(13) There is a saying that the increase of salary is always less than the increase of goods. ***This saying*** reflects the reality in some degree: most of workers are struggling for the food, houses and cars, but it’s hard to accomplish the latter two goals. < Nonnative-TECCL01027.txs>
(14) As is vividly depicted in the material above, the report says that the people who practice square dancing had influenced the neighborhood residents seriously. Thus, ***this issue*** has given rise to a lot of confrontations between them. < Nonnative-TECCL02812.txt>

Taken together, the integrated analysis yields a clear pattern: the descriptive convergence observed in Section 4.2 is statistically robust, while the descriptive divergences are not. Neither the non-significance of *distance* in the L2-only regression nor the absence of deeper interaction splits in the L2-only tree constitutes a statistically reliable group difference when the full dataset is considered. The only divergence that survives statistical scrutiny is the single, localized context—L2 writers’ quantitative overuse of explicit forms in the highest-accessibility topic-continuity context.

## 5. Discussion

The usage-based approach has documented L2 learners’ general convergence with native speakers in the probabilistic constraint patterns governing syntactic alternations. Whether similar dynamics operate at the discourse level, however, has remained an open question. This study addresses this question by examining the abstract anaphoric choice between *this* and *this*+NP in argumentative writing. We analyzed a dataset of 1,304 instances from L2 learners and 1,303 from a native English baseline. Each instance was annotated for discourse-internal factors operationalizing accessibility theory [26,31], which conceptualizes referential choice as a trade-off between clarity and economy [27–29]. We then employed a tripartite multifactorial analysis to statistically quantify constraint strength, ranking, and interactions. Our analysis proceeded in three phases: first, establishing a native (L1) baseline constraint competition model; second, constructing an independent L2 model; and third, conducting a comparative L1-L2 analysis to determine whether the descriptive patterns of convergence and divergence observed in the first two phases are statistically reliable.

Our analysis of the L1 dataset confirms that all four discourse-internal factors significantly constrain the abstract anaphoric choice, consistent with the core tenet of accessibility theory [26,31]. Specifically, native speakers prefer the reduced form *this* in contexts of high accessibility—characterized by given information, non-nominal antecedents, subject position, and previous distance. Conversely, they favor the explicit *this*+NP in contexts of lower accessibility, signaled by new information, nominal antecedents, non-subject position, and longer distance. Crucially, while prior research only identified referential distance as a primary factor [33], our study reveals that accessibility is collectively realized through a suite of interacting discourse-internal factors, thereby substantially expanding the known repertoire of constraints governing this alternation.

Furthermore, our results demonstrate that these factors do not operate in isolation but compete within a probabilistic system, exhibiting a clear hierarchy of influence. This extends the descriptive framework of the accessibility theory by revealing the competition among multiple constraints in shaping referential choice. The observed hierarchy is consistent with the usage-based competition model, which posits that language knowledge reflects probabilistic weights for competing linguistic cues through exposure to language in use [1–5]. Given that demonstrative *this* is fundamentally a device for managing discourse coherence and information structure [14,23], the relative strength of each factor corresponds to its role in this discourse function. The most powerful factor, *givenness*, acts as the primary signal for textual coherence, directly controlling topic continuity or shifts to a new one. It is followed by *antecedent-type*, which relates to conceptual unity. Non-nominal antecedents—such as propositions or events—form semantically cohesive wholes that, because of their richer informational content, may be more readily retrieved from memory, making the reduced form *this* a natural choice [25]. The role of *subjectness*, while less dominant, is grammatically pivotal, as the subject position serves as the canonical site for topic continuity [62]. In stark contrast, *distance*, which pertains to linear proximity rather than core discourse-pragmatic relations, exerts the weakest influence—consistent with its status as a secondary modulator rather than a primary driver of abstract anaphoric choice.

Critically, our analysis revealed systematic interaction patterns that illuminate how these constraints collectively negotiate the trade-off between clarity and economy—a dynamic not captured by accessibility theory. The three factors central to information structure (*givenness*, *antecedent-type*, and *subjectness*) exhibit frequent and complex interactions, as they work in concert to define the fundamental discourse coherence and information structure. In contrast, *distance* demonstrates fewer and weaker interactions, operating more independently as a simple modulator of cognitive load within contexts already defined by the other, more powerful factors. This pattern of interactions provides further, robust evidence that *distance* is a less integral and robust influence on abstract anaphoric choice, thereby challenging its predominance in prior studies [33].

Overall, these findings on the L1 dataset demonstrate how the usage-based approach can account for the observed dynamics of constraint competition. By quantifying the relative weights and interaction patterns of these constraints, our multifactorial analysis moves beyond description of referential outcomes to model the probabilistic relationships that characterize naturalistic production. In doing so, this study extends the competition model’s application, demonstrating that its principles of probabilistic cue-weighting are relevant not only to lexical and syntactic phenomena but also to the domain of abstract discourse.

Our independent L2 analysis revealed strong descriptive convergence with the L1 baseline: learners show sensitivity to the same core constraints, the same constraint hierarchy, and the same fundamental interaction logic. This convergence was subsequently confirmed by the integrated L1-L2 statistical analysis. These findings align with previous research on syntactic alternations in L2 research [6–8,11–13] and are consistent with the usage-based view that L2 learners can develop probabilistic constraint systems through exposure to language in use [2]. Consequently, these findings demonstrate that the usage-based framework, already well-established in L2 research on syntactic alternations, can be extended to the domain of abstract discourse.

The L2-only analysis also revealed minor descriptive divergences: *distance* did not reach significance in the regression, and the L2 learners showed reduced granularity at the deepest interaction levels. One might interpret these patterns as reflecting the weaker status of *distance* as a linguistic cue and the greater difficulty learners face in acquiring the most nuanced usage contexts—precisely the areas where more extensive input is needed to extract probabilistic generalizations from repeated encounters with language. However, the integrated L1-L2 analysis demonstrated that neither the non-significance of *distance* in the L2-only regression nor the absence of deeper interaction splits in the L2-only tree constitutes a statistically reliable group difference when the full dataset is considered. This underscores the importance of moving beyond descriptive comparisons of independently analyzed L1 and L2 models, as apparent differences in usage patterns may not always reflect statistically robust divergences.

The only divergence that survived statistical scrutiny was a single, localized context: L2 learners showed reduced sensitivity to the highest-accessibility topic-continuity context, using the explicit form *this*+NP significantly more than native speakers. This finding aligns with the well-documented phenomenon of overexplicitness in English L2 personal anaphora [15,34–37], indicating that this feature of L2 acquisition extends to the abstract domain. The pattern is consistent with the PPVH [40], which posits that L2 learners, to avoid communicative breakdown, strategically prioritize the principle of clarity while frequently violating the principle of economy. Our results support this account by demonstrating that learners systematically overuse explicit forms not only in personal topic-continuity contexts but also in abstract propositional ones. Consequently, this study validates the extension of the PPVH’s descriptive framework from personal to abstract anaphora.

However, the PPVH remains a descriptive account of the outcome. The usage-based approach addresses this explanatory gap by proposing that the observed overexplicitness emerges from two interconnected factors: entrenchment and statistical learning under input constraints.

First, the usage-based approach posits that language knowledge emerges from exposure to high-frequency, contextually embedded language use [1–5]. Within this view, entrenchment refers to the process by which frequently encountered forms become cognitively solidified as default choices [63]. L2 learners, who typically have less extensive input in the target language, may rely more heavily on such entrenched forms—a tendency that can result in overgeneralizations and simplifications [6,13]. This pattern is evident with our data: The explicit form *this*+NP occurs with higher overall frequency than the reduced *this*. For L2 learners, this high-frequency form therefore becomes the most entrenched, easily accessible “safe” option, leading to its systematic overuse.

Second, from a usage-based perspective, the acquisition of constraints in language alternations is achieved through associative statistical learning, a process whose success is contingent upon a constraint’s ease of processing, among others [20,21,42,43]. Our analysis contributes to understanding this process by demonstrating that the primary challenge for L2 learners lies not merely in acquiring individual constraints, but in mastering their probabilistic interactions. Our multifactorial analysis, operationalized through conditional inference trees, reveals the precise conditions of this deficit: the complex convergence of *givenness*, *antecedent-type*, and *subjectness* that defines a prototypical high-accessibility abstract propositional topic continuity context. For L2 learners with less robust input, the subtle probabilistic cue that this context licenses a reduced form results in a weak form-function mapping. Consequently, they default to the entrenched, transparent form (*this*+NP) to mitigate processing costs, resulting in the systematic overexplicitness we observed. Thus, we move beyond identifying that learners struggle to explain when and why they struggle—namely, with the complex statistical landscape of naturalistic discourse.

Before concluding, we consider several alternative explanations for the observed patterns.

One alternative explanation concerns the potential influence of prescriptive instruction. As one reviewer noted, English learners are often explicitly taught to avoid bare *this* in academic writing [24]. Such instruction may create a bias toward explicit forms. However, if prescriptive rules were the primary driver, we would expect learners to overuse *this*+NP across most discourse contexts—yet they demonstrated target-like sensitivity in five of six contexts. The divergence is specifically localized to the highest-accessibility topic-continuity context, where the reduced form is most natural for native speakers. This suggests that while prescriptive instruction may reinforce learners’ tendency toward explicit forms, it does not override their broader implicit sensitivity to the constraint system.

Another possibility concerns the role of L1 transfer. Mandarin possesses a parallel demonstrative system (*zhe* vs. *zhe* + NP) [44,45], making positive transfer a plausible contributor to learners’ overall success. However, a simple transfer account would predict identical L1-L2 performance—a prediction our data do not support. Learners overuse the explicit form despite having a parallel L1 structure, suggesting transfer alone cannot explain the pattern. While subtle transfer effects cannot be entirely ruled out, the overall pattern is more consistent with usage-based accounts emphasizing input frequency and statistical learning.

In summary, this study provides evidence that L2 learners can develop probabilistic constraint systems for abstract anaphora that are broadly similar to those of native speakers. The observed divergence in topic-continuity context is not indicative of a fundamentally different underlying system but rather reflects differences in input conditions that affect the statistical learning of specific, highly demanding discourse context. Importantly, what this study demonstrates is convergence in outcome: L2 learners exhibit a constraint system structurally similar to that of native speakers. This outcome is consistent with usage-based predictions about statistical learning from input, though it does not directly speak to the developmental paths by which learners arrive at this endpoint.

By integrating the descriptive power of the accessibility theory and the PPVH with the explanatory framework of the usage-based approach, this study accounts for both the patterns of convergence observed across most discourse contexts and the specific conditions under which divergence emerges. The findings thus extend the applicability of the usage-based competition model from lexical and syntactic phenomena to the domain of abstract discourse.

## 6. Conclusion

This study has shown that the principles of the usage-based approach to L2 research, previously established for syntactic alternations, extend to the domain of abstract discourse. Through a three-phase multifactorial analysis of the anaphoric choice between *this* and *this*+NP, we have provided a probabilistic model of how this choice is constrained by a hierarchy of competing discourse-internal factors in L1 production. The independent L2 analysis revealed that learners show sensitivity to the same core constraints, constraint hierarchy, and fundamental interaction logic as native speakers, and statistical comparison confirmed broad convergence between the two groups. The only reliable divergence was localized to a single context: L2 learners overused the explicit *this*+NP in the highest-accessibility topic-continuity context. This pattern can be understood as arising from the interaction of entrenchment and statistical learning under the specific conditions of limited L2 input, rather than reflecting a fundamentally different underlying constraint system.

Several limitations of the present study point to valuable directions for future research. First, while our focus on Mandarin learners minimizes the likelihood of overt morphosyntactic transfer, more subtle discourse-level transfer effects cannot be entirely ruled out. Disentangling statistical learning from L1 transfer would require comparable Mandarin production data and a systematic comparison with learners from diverse L1 backgrounds. Future research including such populations could reveal how different types of transfer—from overt morphosyntactic parallels to subtle discourse-pragmatic conventions—interact with the development of English abstract anaphora.

Second, our dataset, while substantial, did not permit a granular analysis of the effect of L2 proficiency and therefore cannot reveal the developmental trajectories by which probabilistic sensitivity emerges or how constraint hierarchies evolve with increasing proficiency. Future longitudinal research incorporating proficiency as a continuous variable could clarify whether sensitivity develops gradually or exhibits threshold effects, and whether overexplicitness decreases with advancing proficiency.

Finally, corpus-based studies like this one are valuable for revealing patterns in end-state production, but they cannot directly adjudicate questions about the mechanisms by which such knowledge is acquired. As reviewers noted, end-state production data alone cannot resolve debates about whether L1 and L2 acquisition rely on shared learning mechanisms. Complementary experimental methods are needed to address these questions. Follow-up studies employing controlled rating tasks or felicity judgments could probe learners’ implicit knowledge of the discourse conditions licensing each variant, offering insight into the representations that underlie production choices. By integrating corpus analysis with psycholinguistic experimentation, future research can work toward a more complete understanding of how L2 learners navigate discourse-level phenomena.

## Supporting information

S1 File Metadata. (XLSX)

S1 Code Plosone-code. (R)
